# In Vivo Imaging of Anterograde and Retrograde Axonal Transport in Rodent Peripheral Nerves

**DOI:** 10.1007/978-1-0716-0585-1_20

**Published:** 2020-01-01

**Authors:** James N. Sleigh, Andrew P. Tosolini, Giampietro Schiavo

**Keywords:** Intravital imaging, Molecular motor, Motor neuron, Neurodegeneration, Neuromuscular disease, Neuropathy, Neurotrophin, Sensory neuron, Sciatic nerve, Tetanus neurotoxin

## Abstract

Axonal transport, which is the process mediating the active shuttling of a variety cargoes from one end of an axon to the other, is essential for the development, function, and survival of neurons. Impairments in this dynamic process are linked to diverse nervous system diseases and advanced ageing. It is thus essential that we quantitatively study the kinetics of axonal transport to gain an improved understanding of neuropathology as well as the molecular and cellular mechanisms regulating cargo trafficking. One of the best ways to achieve this goal is by imaging individual, fluorescent cargoes in live systems and analyzing the kinetic properties of their progression along the axon. We have therefore developed an intravital technique to visualize different organelles, such as signaling endosomes and mitochondria, being actively transported in the axons of both motor and sensory neurons in live, anesthetized rodents. In this chapter, we provide step-by-step instructions on how to deliver specific organelle-targeting, fluorescent probes using several routes of administration to image individual cargoes being bidirectionally transported along axons within the exposed sciatic nerve. This method can provide detailed, physiologically relevant information on axonal transport, and is thus poised to elucidate mechanisms regulating this process in both health and disease.

## 1 Introduction

Axonal transport is the indispensable process whereby molecular motor proteins actively traverse the microtubule network of neurons to deliver a variety of different cargoes from one end of an axon to the other. Kinesin family proteins drive anterograde transport from the cell body to the axon tip, whereas retrograde transport is contingent upon cytoplasmic dynein [1]. Mutations in several genes essential to this bidirectional trafficking are known to cause neurodevelopmental and neurodegenerative conditions [2, 3], while its disturbance is thought to underlie, or at least contribute to, a broad variety of neurological disorders, including amyotrophic lateral sclerosis (ALS), Alzheimer’s disease, and Char-cot–Marie–Tooth disease [4]. It is therefore essential that this dynamic process is studied in relevant models in order to fully determine the consequences of impaired axonal transport on neuronal dysfunction and disease.

Real-time tracking of individual cargoes in live axons has long been reported in various primary neuronal cultures and ex vivo tissue preparations; however, there is evidence to indicate that these experimental environments do not always fully replicate what is observed in intact neurons in vivo [5–9]. This may have a plethora of causes, for instance perturbed physical and chemical interactions with neighboring cells [10], altered activity [11], and insufficient maturation [12], which can only be convincingly overcome by studying transport in vivo. Several methods have been developed to study axonal transport en masse in live rodents [13, 14]; however, these noninvasive techniques have limited temporal and spatial resolution and can be impacted by neuronal death. Although caveats apply, intravital imaging of axonal transport of individual cargoes is likely to provide more physiologically relevant insights into the process, while permitting detailed, real-time dissection of cargo dynamics [15].

We have previously published a method to assess in vivo axonal transport of signaling endosomes in peripheral nerve axons of the sciatic nerve in live, anesthetized rodents [6]. Neurotrophin-containing signaling endosomes are visualized by intramuscular injection of one of two fluorescently labeled probes: an atoxic binding fragment of tetanus neurotoxin (H_C_T) or an antibody against the extracellular domain of p75 neurotrophin receptor (p75^NTR^). These probes are internalized at the neuromuscular junction and afferent nerve endings before being retrogradely transported within signaling endosomes toward the cell body of motor and sensory neurons of the sciatic nerve [16]. We have used this method to show that mutant SOD1^G93A^ and TDP-43^M337V^ mice modelling ALS display early deficits in signaling endosome transport in vivo [17, 18], which can be rescued in SOD1^G93A^ mice by p38 MAPK [19] or IGF1R inhibition [20]. Indicating that neurodegeneration is not always linked to disturbances in transport, we have shown that endosome trafficking remains unaffected in Fus^Δ14/+^ ALS mice, spinal and bulbar muscular atrophy mice, and aged wild-type animals [18, 21, 22].

This chapter provides an updated and improved, step-by-step description of the technique to image bidirectional transport of multiple different cargoes in rodent sciatic nerves in vivo. We focus on mice throughout, but the protocol can be adapted for use with rats. We begin by detailing the injection of fluorescent probes into different muscles of the lower leg, followed by exposure of the sciatic nerve for imaging of signaling endosomes. We also outline an extension to the method that permits the visualization of additional cargo types (e.g., mitochondria) through injection of lipophilic dyes directly into the sciatic nerve [23], obviating the requirement for genetically encoded, fluorescent reporter strains (e.g., “MitoMice” [24]). This permits the simultaneous labeling of different cargoes with distinct fluorophores, which will be particularly useful for developing a broader understanding of transport, as the trafficking of different cargoes is differentially regulated [25]. The method reported here will not only aid the assessment of axonal transport in rodent models of neurological disease, it can be used to determine the impact of aging and different drug treatments (e.g., chemotherapeutic molecules) on cargo trafficking, as well as improving our understanding of the basic mechanisms regulating the process.

## 2 Materials

### 2.1 Intramuscular Injection of Fluorescent Probes

#### 2.1.1 Equipment

1. Vortex mixer.
2. Desktop centrifuge.
3. Heating pad.
4. Surgery/operating microscope.
5. Isoflurane vaporizer/anesthesia machine with induction chamber and mask stabilizer.
6. Weighing scales (optional).
7. Hair clippers.
8. Fine straight forceps.
9. Small spring scissors.
10. Hamilton microliter syringe (701 N, volume 10 μl, needle size 26 s ga, bevel tip, needle L 51 mm).
11. Micropipette puller.
12. Dissecting microscope.
13. Fine curved forceps.

#### 2.1.2 Supplies

1. Fluorescently labeled H_C_T [6].
2. Fluorescently labeled anti-p75^NTR^ [6].
3. Recombinant human/murine/rat brain-derived neurotrophic factor.
4. Phosphate buffered saline (PBS): 137 mM NaCl, 10 mM Na_2_HPO_4_, 2.7 mM KCl, 1.8 mM KH_2_PO_4_–HCl, pH 7.4.
5. 0.2 ml PCR tube.
6. Aluminum foil.
7. Surgical drape.
8. Isoflurane.
9. Paper towels.
10. Surgical tape.
11. Eye lubricant.
12. Cotton swab.
13. 70% (v/v) ethanol in distilled water.
14. Surgical suture.
15. Graduated, glass micropipette with microliter markings and plunger (Drummond Scientific, 5-000-1001-X10).
16. Scalpel blade (optional).

### 2.2 Sciatic Nerve Exposure

#### 2.2.1 Equipment

1. Heating pad.
2. Isoflurane vaporizer/anesthesia machine with induction chamber and mask stabilizer.
3. Hair clippers.
4. Small spring scissors.
5. Fine straight forceps.
6. Fine curved forceps.
7. Scissors.

#### 2.2.2 Supplies

1. Paper towels.
2. Isoflurane.
3. Surgical tape.
4. Cotton swab.
5. 70% (v/v) ethanol in distilled water.
6. Saline: 0.9% w/v NaCl in distilled water.
7. Cotton wool.
8. Magic tape (Scotch) or Parafilm.

### 2.3 Sciatic Nerve Injection

#### 2.3.1 Equipment

1. Vortex mixer.
2. Desktop centrifuge.
3. Isoflurane vaporizer/anesthesia machine with induction chamber and mask stabilizer.
4. Heating pad.
5. Dissecting microscope.
6. Fine curved forceps.

#### 2.3.2 Supplies

1. 0.2 ml PCR tube.
2. Tetramethylrhodamine, ethyl ester, perchlorate (TMRE).
3. PBS.
4. Bromophenol blue (optional).
5. Aluminum foil.
6. Isoflurane.
7. Graduated, glass micropipette with microliter markings and plunger (Drummond Scientific, 5-000-1001-X10).
8. Saline.
9. Cotton wool.

### 2.4 In Vivo Imaging of Axonal Transport

#### 2.4.1 Equipment

1. Customized microscope stage (45 × 18 mm window for glass coverslip).
2. Oil immersion 40 × or 63× objective.
3. Inverted confocal microscope with environmental chamber.
4. Computer with microscope control and image acquisition software.

#### 2.4.2 Supplies

1. Surgical or masking tape.
2. Glass coverslip (22 × 64 mm, thickness no. 1).
3. Immersion oil for fluorescent imaging at 37 °C.
4. Saline.

## 3 Methods

### 3.1 Intramuscular Injection of Fluorescent Probes

#### 3.1.1 Fluorescent Probe Preparation

5 μg of fluorescently labeled H_C_T or 1 μg of fluorescently labeled anti-p75^NTR^ is coinjected with 25 ng of recombinant BDNF per muscle in a volume of 1–2 μl of PBS depending on the muscle (*see* **Note 1**). H_C_T and anti-p75^NTR^ target overlapping, but distinct, populations of neurons and can be used to answer different experimental questions (*see* **Note 2**). For simplicity and because we use it routinely, we will focus for the remainder of this protocol on using H_C_T for in vivo labeling of signaling endosomes.

1. To prepare H_C_T for injection into the gastrocnemius and tibialis anterior muscles (*see* **Note 3**), dilute fluorescently labeled H_C_T to 2.5 μg/μl and recombinant BDNF to 12.5 ng/μl in 6 μl of sterile PBS in a 0.2 ml PCR tube. Injection of 2 μl of this solution into each of the two muscles will result in the desired amounts of each substance being delivered. Excess solution is made due to the small volumes being pipetted and injected.
2. To prepare H_C_T for soleus muscle injection (*see* **Note 3**), mix the same reagents to generate 3 μl of solution with 5 μg/μl of fluorescently labeled H_C_T and 25 ng/μl of recombinant BDNF. Soleus muscles are smaller and therefore must be injected with a reduced volume (1 μl) of more concentrated solution to achieve similar amounts of protein injection.
3. Vortex the solution to mix and spin down using a desktop centrifuge, before protecting from light by wrapping in aluminum foil and leaving on ice. Solutions for injection should be prepared just before use and not freeze-thawed.

#### 3.1.2 Fluorescent Probe Injection into the Gastrocnemius and Tibialis Anterior Muscles

All mouse handling and experiments must be performed in accordance with local ethical guidelines. The work involving animals presented in this chapter was conducted under license from the UK Home Office in accordance with the Animals (Scientific Procedures) Act (1986), and approved by the University College London (UCL)—Institute of Neurology Ethics Committee.

**Fig. 1:**
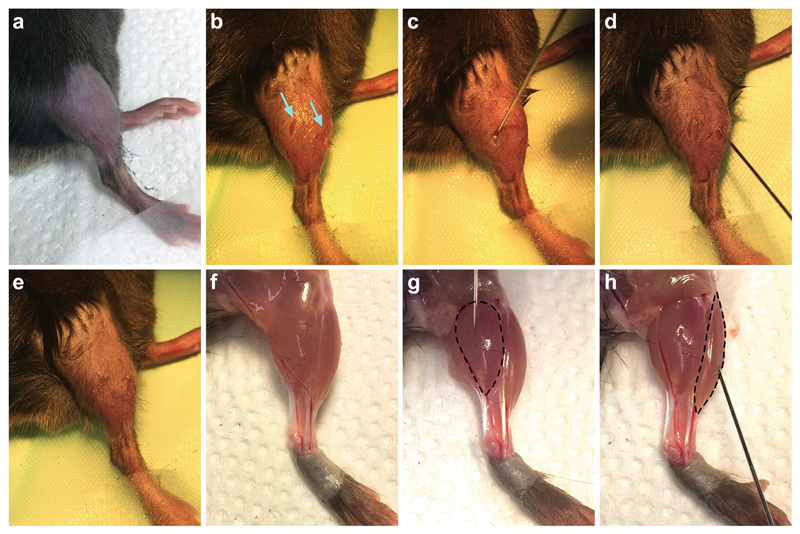
Injection of fluorescent probes into the gastrocnemius and tibialis anterior muscles. (**a**) Using clippers, the fur covering the lateral aspect of the right lower limb overlaying the gastrocnemius and tibialis anterior muscles is removed. (**b**) The hind paw is then taped to a surgical drape, the shaved fur dabbed with an ethanol-soaked cotton swab, and two small (1–2 mm), vertical incisions (cyan arrows) cut through the skin above both muscles. (**c**, **d**) The fluorescent probe (H_C_T or p75^NTR^ antibody) is then injected into the gastrocnemius (**c**) and tibialis anterior (**d**) muscles using a Hamilton microliter syringe. (**e**) The incisions are closed with single perpendicular stitches before allowing the animal to recover. Steps **a–e** are performed under isoflurane-induced anesthesia and surgical conditions. (**f**) To aid understanding of lower leg musculature, the skin has been removed from a culled mouse. (**g**, **h**) The lateral gastrocnemius (**g**) and tibialis anterior (**h**) muscles (highlighted by dashed lines) are found on the dorsal and ventral aspects of the leg, respectively. Note that the gastrocnemius is injected at a steep angle from the plane of the table (almost directly from above), whereas the tibialis anterior is injected from a much shallower, almost horizontal angle from the direction of the hind paw. To aid visualization, the biceps femoris muscle has been removed in panels g and h. *See* Subheading 3.1.2

1. Prepare for surgery by (a) turning on the heating pad to body temperature, which helps to minimize heat loss during surgery, and covering it with a surgical drape, (b) switching on and focusing the operating microscope, and (c) unpacking the presterilized tools, suture, saline, cotton swab, ethanol, surgical tape, and fluorescent probe onto the edge of the drape.
2. Check that the isoflurane vaporizer/anesthesia machine has sufficient isoflurane for the duration of surgery, and top up if necessary. Switch on the machine, ensure an oxygen flow rate of 1–2 l/min, and anesthetize the animal with isoflurane (4–5% in O_2_) in the induction chamber lined with paper towels.
3. Once sufficiently anesthetized (absence of righting reflex and slowed, steady breathing rate), move the mouse to the mask stabilizer on a paper towel and continue anesthesia at a reduced concentration of isoflurane (2–4% in O_2_). As the animal is immobilized, this is a convenient point for weighing, if required.
4. Using hair clippers, cut the fur from skin covering the gastroc-nemius and tibialis anterior muscles of the lower limb on one side of the mouse (Fig. 1a). We usually inject the right side of the body, but the left works equally well. Remove the clipped hair using surgical tape.
5. Apply eye lubricant with a cotton swab to restrict drying of the eyes during surgery, and move the mouse in the mask stabilizer to the surgical drape, lying it on its left side with the mask furthest away from you. Extend the right leg toward you (away from the anesthesia mask), and tape the top of the hind paw to the surgical drape. Doing this slightly twists the limb aiding the injections.
6. Using a cotton swab, dab the shaved area with 70% ethanol to reduce fur contamination and maintain sterility.
7. Before making any incisions, confirm that the level of anesthesia remains sufficient by pinching the toes of the hind paws and checking that the pedal withdrawal is absent. Breathing depth and rate also serve as complementary indicators. Careful and continual checking of these signs must occur throughout all described surgical procedures to ensure a stable and sufficient depth of anesthesia.
8. Using fine straight forceps to gently clasp the skin, make two small (1–2 mm), vertical incisions, one above both the gastrocnemius and tibialis anterior muscles, with small spring scissors held perpendicular to the forceps (Fig. 1b).
9. Once the muscles have been clearly exposed, take up the solution containing the fluorescent probe into the microsyringe, and slowly inject 2 μl into each muscle (in any order). Ensure that the eye of the needle is within the muscle before injecting and leave the microsyringe in the muscle for 3–5 s before slowly removing to restrict leakage of the probe (*see* **Note 4**). Inject the lateral head of the gastrocnemius using a steep angle from the plane of the table (Fig. 1c) and use a much shallower, almost horizontal, angle (i.e., 10–20°) for the tibialis anterior (Fig. 1d) (*see* **Note 5**).
10. Close each incision with a small, perpendicular stitch using a surgical suture (Fig. 1e), before placing the animal back in the cage for recovery and monitoring. We do not apply analgesics because they can influence axonal transport [26, 27]. Depending on how long the surgery takes, animals usually recover from this procedure within 5–15 min but should be regularly checked postsurgery. When mastered, the surgical part of this protocol (Subheading 3.1.2) should take 5–10 min per mouse. To aid understanding and improve injections, the lower leg anatomy should be studied (Fig. 1f–h).

#### 3.1.3 Fluorescent Probe Injection into the Soleus Muscle

**Fig. 2:**
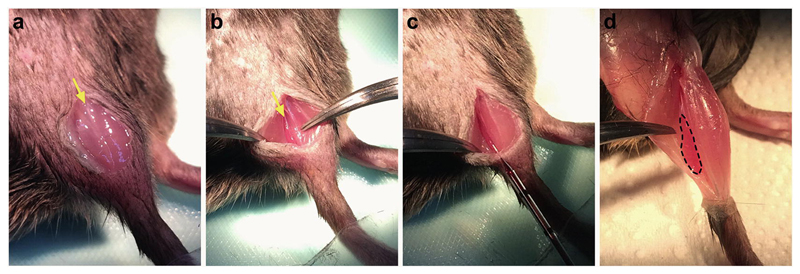
Injection of fluorescent probes into the soleus muscle. (**a**) A vertical incision of 6–8 mm is made in the shaved skin overlaying the lateral aspect of the right hind limb. To locate the soleus muscle, the connective tissue between the gastrocnemius and tibialis anterior muscles (yellow arrow) is carefully cut and the two muscles are separated without damaging their fibers. (**b**) The soleus muscle (yellow arrow), which is darker in appearance, is exposed using forceps by gently pulling back the gastrocnemius. (**c**) The fluorescent probe is injected into the soleus using a pulled, glass micropipette, before closing the skin incision using 2–4 stitches. Note that the injection angle is shallow, like that of the tibialis anterior injection. Steps **a–c** are performed under isoflurane-induced anesthesia and surgical conditions. (**d**) The soleus muscle (highlighted by dashed line) is found on the dorsal aspect of the leg, nestled within the gastrocnemius, as shown after skin removal from a dead mouse. *See* Subheading 3.1.3

1. Before preparing the fluorescent probe solution (*see* Subheading 3.1.1), pull the graduated, glass micropipettes with a micropipette puller (*see* **Note 6**) [28]. Using a dissecting microscope, carefully break the tip of the micropipette using fine straight forceps to allow pipetting.
2. At the incision stage of the above protocol (*see* **step 8** in Subheading 3.1.2), instead of making two small cuts, make a single vertical incision of 6–8 mm in the shaved skin overlaying the lateral aspect of the lower hind limb (Fig. 2a).
3. Locate the soleus muscle by carefully cutting the connective tissue running between the gastrocnemius and tibialis anterior muscles (Fig. 2a) using small spring scissors or a scalpel blade, and gently pull back the surrounding muscles using fine curved forceps (Fig. 2b). The soleus muscle is easy to identify due to its darker appearance (Fig. 2b–d).
4. Using the glass micropipette, slowly inject 1 μl of the probe into the soleus muscle, using a shallow angle of injection from the plane of the table (i.e., 10–20°) (Fig. 2c and *see* **Note 5**). Leave the micropipette in the muscle for 3–5 s to restrict probe seepage.
5. Close the incision with 2–4 individual stitches (depending on incision length) using a surgical suture and allow the mouse to recover as detailed above (*see* **step 10** in Subheading 3.1.2). Once again, practice dissecting the hind limb muscles from a dead mouse in order to better understand where the soleus muscle is located (Fig. 2d). When mastered, this part of the protocol should take 5–10 min per mouse.

### 3.2 Sciatic Nerve Exposure

**Fig. 3:**
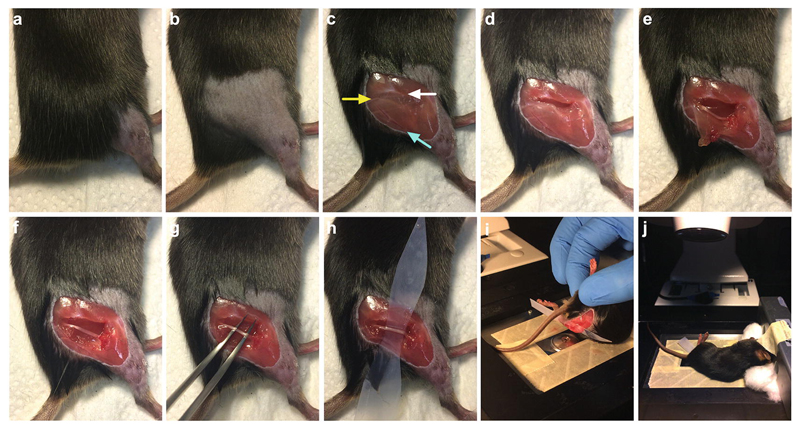
Exposure of the sciatic nerve for in vivo imaging. (**a**, **b**) The fur overlaying the right thigh region is clipped. (**c**) Ethanol-dampened skin covering the thigh is removed, revealing the underlying musculature. The sciatic nerve (yellow arrow) can be observed beneath the femur (white arrow). The superficial blood vessel (cyan arrow), running from the hip region down toward the gastrocnemius, provides a useful guide for muscle removal. (**d**) A 6–10 mm horizontal incision, parallel with the femur, is made in the connective tissue/muscle between the femur and the sciatic nerve. (**e**) A perpendicular incision through muscle is made from the femur toward the superficial blood vessel, over the region where the sciatic nerve bifurcates into the tibial and common fibular/peroneal nerves. (**f**) A final cut through muscle, adjacent to the blood vessel, is made toward the initial incision site near the hip, creating a triangular window framing the exposed sciatic nerve. (**g**) The sciatic nerve is doused in saline before separating the nerve from the underlying muscle by carefully placing forceps underneath the nerve to displace the connective tissue. (**h**) Folded magic tape, cut to a violin-like shape with a pointed end and width of 3–6 mm, is then slid beneath the nerve to separate it from surrounding tissue, thus aiding imaging. (**i**, **j**) The mouse is placed on the stage of an inverted confocal microscope with its environmental chamber prewarmed to 37 °C. The body weight of the animal is holding the sciatic nerve in place on the glass coverslip above the objective lens. All steps are performed under isoflurane-induced anesthesia. *See* Subheading 3.2

1. At least 1 h before in vivo imaging of the sciatic nerve (*see* Subheading 3.4 below), turn on the environmental chamber surrounding the inverted confocal microscope and set it to 37 °C; this will allow sufficient time for the temperature to equilibrate. Body temperature can have a significant impact on transport [29] (and our own observations); thus, it is imperative that the temperature of the imaging chamber is carefully regulated.
2. 4–8 h post-intramuscular injection (*see* **Note 7**), turn on and allow the heating pad to warm up, and cover it with paper towels.
3. Reanesthetize the injected mouse (*see* Subheading 3.1) with isoflurane as above (*see* **steps 2** and **3** in Subheading 3.1.2), and shave the fur covering the right leg and hip region (Fig. 3a, b), removing any excess fur with surgical tape.
4. Place the mouse in the anesthetic mask lying on its left side and use tape on top of the right hind paw to stick the leg down to a paper towel. Provide a small amount of tension in the sciatic nerve to aid dissection by sticking the hind paw more perpendicular to the body, rather than in the same plane as that in **step 5** of Subheading 3.1.2.
5. After using a cotton swab to dampen the fur with 70% ethanol, remove the skin overlaying the thigh region with small spring scissors (Fig. 3c). At this point, the sciatic nerve can be seen through connective tissue and muscle, inferior to the femur, and superior to a superficial blood vessel that runs from the thigh region toward the gastrocnemius.
6. In the region of the hip, make an incision with spring scissors in the connective tissue between the femur and sciatic nerve, taking care not to cut or touch the nerve (Fig. 3d). It can help to use forceps in one hand to grasp the connective tissue and make the initial cut with the other.
7. Cut along the level of the femur for 6–10 mm from the hip region to toward the knee and stop in the location where the sciatic nerve begins to branch into the tibial and common fibular/peroneal nerves.
8. Start cutting the musculature at an angle perpendicular to the original incision, until the superficial blood vessel is reached (Fig. 3e). Cutting this vessel does not prevent imaging of axonal transport, but blood loss can weaken the animal and obscure the sciatic nerve during imaging. If this does occur, wash away the blood with saline and try to staunch the flow with cotton wool before proceeding.
9. Continue to cut along the blood vessel up toward the hip, until the original incision is reached. This should result in the removal of a triangular piece of muscle that was originally overlaying the sciatic nerve (Fig. 3f).
10. Apply saline to the sciatic nerve before using fine curved forceps to dislodge the connective tissue holding the sciatic nerve in place. This is done by pushing the closed forceps under the nerve and slowly moving from side to side (Fig. 3g). The application of saline provides lubrication to the nerve and restricts dissection tools from sticking to the epineurium. Be gentle with the nerve and limit forceful contact; for example, do not clasp the nerve between the forceps, because physical damage (e.g., nerve crush) may impact axonal transport.
11. Double up some magic tape by folding in half and with scissors cut to the shape of a violin with a pointed end and a flat end. Alternatively, Parafilm can be used without folding. This tape will separate the nerve from the underlying tissue to aid imaging. At its two widest points the violin should be 4–6 mm— broad enough to prevent slippage out from the nerve, but not too wide that it cannot fit under—and at its narrow mid-point should be 3–4 mm (the dimensions depending on size of the mouse). The tape should be about 5 cm in length; if much longer, it can fold and impair imaging, and if much shorter, it is more likely to slip out from behind the nerve.
12. Thread the violin-shaped magic tape under the sciatic nerve, pointed-end first, until it rests with the nerve overlying the narrow part in the middle of the tape (Fig. 3h). Recut if too wide to fit and do not force as this may damage the nerve.
13. The surgical procedure should take 10–15 min.
14. If cargoes in addition to signaling endosomes are to be simul-taneously imaged (e.g., mitochondria), then proceed to Subheading 3.3. If signaling endosomes are the only cargoes to be imaged, place some saline-soaked cotton wool on top of the sciatic nerve, move the mouse to the anesthetic chamber placed on a heating pad, and proceed directly to Subheading 3.4.

### 3.3 Sciatic Nerve Injection

**Fig. 4:**
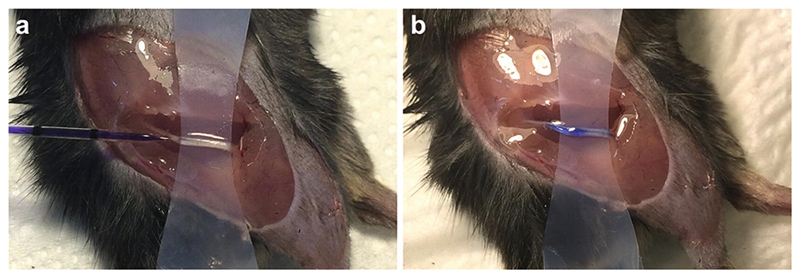
Injection of fluorescent probes into the sciatic nerve. (**a**) Fluorescent reporter dyes, such as mitochondria-targeting TMRE, can be injected directly into the sciatic nerve using a pulled, glass micropipette. The sharp end of the micropipette is carefully inserted beneath the epineurium in the same direction as the nerve by piercing the exterior of the sciatic nerve at a 30–45° angle from the table plane, and then reducing the angle between the nerve and the micropipette to ≈10° upon insertion. Curved forceps can be placed under the nerve to balance the micropipette and aid injection. (**b**) The dye is then slowly injected into the space within the epineurium, facilitating access to individual axons. A dye, such a bromophenol blue, can be included with the fluorescent dye to aid confirmation of successful injection. Sciatic nerve injections are performed under isoflurane-induced anesthesia. *See* Subheading 3.3.

1. This procedure can be combined with Subheading 3.2, omitting Subheading 3.1, if imaging of signaling endosomes is not required.
2. Prior to Subheading 3.2, prepare the fluorescent dye of choice for injection in a 0.2 ml PCR tube (e.g., 2 μm TMRE in PBS) to label active mitochondria (*see* **Note 8**). 0.01–0.1% (w/v) bromophenol blue can be added to the fluorescent probe to aid confirmation of successful injection.
3. Vortex the solution to mix and spin down using a desktop centrifuge, before protecting from light by wrapping in aluminum foil and leaving on ice. Solutions for injection should be prepared just before use and not freeze-thawed.
4. After sciatic nerve exposure and separation from surrounding tissue with magic tape (*see* Subheading 3.2), load a glass micro-pipette with at least 3 μl of fluorescent dye-containing solution (*see* **step 1** in Subheading 3.1.3 for micropipette preparation).
5. Under a dissecting microscope, at a 30–45° angle from the plane of the table, and in line with the sciatic nerve, insert the tip of the graduated glass micropipette into the proximal end of the nerve through the epineurium (Fig. 4a) (*see* **Note 9**).
6. As the tip is inserted, continue to advance into the nerve while reducing the angle from the plane of the table to ≈10°. This allows for more of the micropipette to be inserted without going straight through the sciatic nerve and limiting axon damage (*see* **Note 10**).
7. Gently inject 1–2 μl (depending on body size) of the fluorescent dye into the space within the epineurium (Fig. 4b).
8. The injection procedure should take no longer than 5 min.
9. Place some saline-soaked cotton wool on top of the sciatic nerve to restrict drying, move the animal to the anesthetic chamber placed on the heating pad, and incubate for a minimum of 15 min to permit dye permeation. As an alternative to injection, we have attempted permeation of the nerve by dousing with dye and covering with dye-soaked cotton wool, albeit without success.

### 3.4 In Vivo Imaging of Axonal Transport

**Fig. 5:**
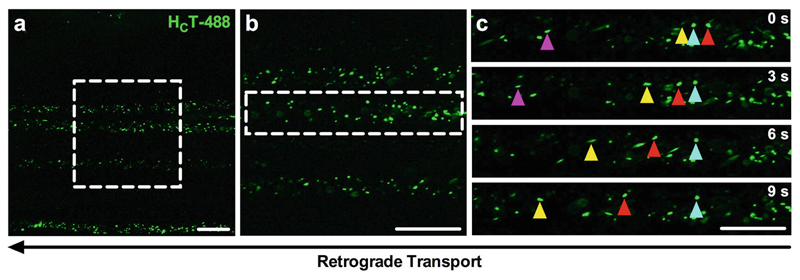
H_C_T is retrogradely transported within signaling endosomes along peripheral nerve axons. (**a**, **b**) Representative images of the sciatic nerve using a 63 × objective without (**a**) and with optical zoom to 100 × (**b**), showing endosomes labeled with H_C_T-488 (green). The dashed boxes in panel a and b represent the imaging regions of panels **b** and **c**, respectively. (**c**) Series of time-lapse confocal microscopy images taken every 3 s (progressing from top to bottom) depicting retrograde transport of H_C_T in sciatic nerve axons. Individual endosome cargoes (e.g., colored triangles) loaded with H_C_T can be tracked across multiple, consecutive images to assess transport dynamics. For all images, retrograde transport is from right to left, and scale bars = 20 μm. *See* Subheading 3.4

**Fig. 6:**
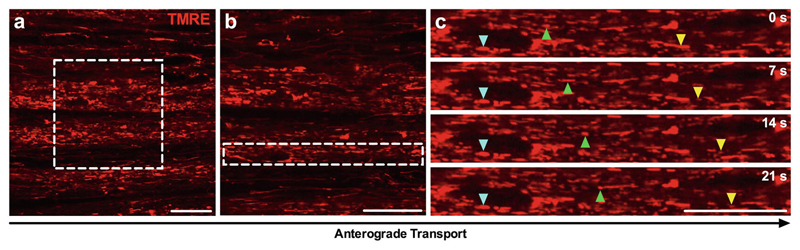
TMRE-labeled mitochondria are bidirectionally transported along peripheral nerve axons. (**a**, **b**) Representative images of the sciatic nerve using a 63 × objective without (**a**) and with optical zoom to 100 × (**b**), showing mitochondria labeled with TMRE (red) by sciatic nerve injection. The dashed boxes in panel **a** and **b** represent the imaging region of panels **b** and **c**, respectively. (**c**) Series of time-lapse confocal microscopy images taken every 7 s (progressing from top to bottom) depicting axonal transport of TMRE- loaded mitochondria in sciatic nerve axons. Mitochondria (e.g., colored triangles) can be tracked across multiple, consecutive images to assess transport dynamics. For all images, anterograde transport is from left to right, and scale bars = 20 μm. *See* Subheading 3.4

**Fig. 7:**
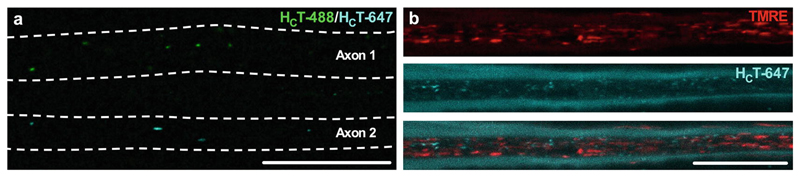
Nerves innervating different muscles, and multiple cargo types can be simultaneously imaged. (**a**) H_C_T labeled with different fluorophores can be injected into multiple muscles of the lower leg to allow for simultaneous imaging and comparison of signaling endosome transport dynamics in axons innervating different muscles, (e.g., H_C_T-488 [green, Axon 1] into the gastrocnemius and H_C_T-647 [cyan, Axon 2] into the tibialis anterior). Dashed, white lines highlight margins of two axons containing H_C_T, which are surrounded by additional, nonhighlighted axons. (**b**) Transport of multiple cargo types can be simultaneously imaged in single axons (e.g., mitochondria [TMRE, red, top panel] and signaling endosomes [H_C_T-647, cyan, middle panel]). The bottom panel represents a merged image of TMRE and H_C_T-647. Scale bars = 20 μm. *See* Subheading 3.4

1. Use surgical or masking tape to secure a glass coverslip to the customized microscope stage, apply immersion oil to the microscope objective (e.g., 40 × or 63×), and connect the stage to the inverted confocal microscope (Fig. 3i). Advance the oil-covered objective until it contacts the glass coverslip (*see* **Note 11**).
2. Feed the anesthetic mask into the environmental chamber surrounding the confocal microscope. Secure the mask onto the microscope stage using surgical tape.
3. Remove the cotton wool from the sciatic nerve and move the mouse from the anesthetic chamber to the mask, laying the animal on its right side with the exposed sciatic nerve in the region of the objective (Fig. 3i). Use a small piece of tape to secure the mouse to the anesthetic mask, but take care not to restrict air flow; this step prevents the mouse from slipping out of the mask while imaging.
4. Add a small amount of saline on top of the coverslip to aid imaging and restrict sciatic nerve desiccation, before carefully aligning the nerve on the coverslip with the objective beneath (Fig. 3i, j).
5. Using the eyepiece, locate the nerve by moving the stage and altering the focal plane (*see* **Note 12**), and identify a region containing axons with labeled cargoes (*see* **Note 13**).
6. If breathing of the animal is causing the sciatic nerve to move too much within the field of imaging, then the mouse will have to be rearranged on the stage, and the nerve relocated (*see* **Note 14**). Maintaining a stable level of anesthesia will help this operation.
7. Using the software controlling the microscope, digitally increase the magnification (e.g., 80–100×) and rotate the field of view so that axons run horizontally from left to right (Figs. 5a, b and 6a, b).
8. Chose an individual axon or multiple adjacent axons for imaging (*see* **Note 15**), and digitally select them using a rectangular field of view of ≈100 ×20 μm for a 40 × objective and ≈60 × 12 μm for a 63 × (Figs. 5c and 6c).
9. Using a maximum laser power of 2% (but preferrably 1%) to limit phototoxicity and photobleaching (*see* **Note 16**), obtain sequential images of the selected region at an optimal resolution. 100–1500 images over 5–20 min can be acquired using a variety of different settings, which are application- and analysis method-specific (*see* **Note 17**).
10. When simultaneously imaging more than one fluorophore (e.g., for different cargo types) (Fig. 7), use dyes with nonoverlapping or limited excitation and emission spectra. This will permit concurrent imaging of fluorophores using the same detector, maintaining an efficient image acquisition time.

Strive to image a minimum of ten trackable cargoes in at least three different axons per animal within an imaging time frame (i.e., from first attempting to visualize the sciatic nerve) of 1 h before culling. We have confirmed that signaling endosome dynamics do not change during this extended imaging period. We perform this protocol as a terminal procedure, but there is scope for recovery and reimaging for longitudinal studies [30], for example, through addition of an imaging window.

### 3.5 Applicability

**Fig. 8:**
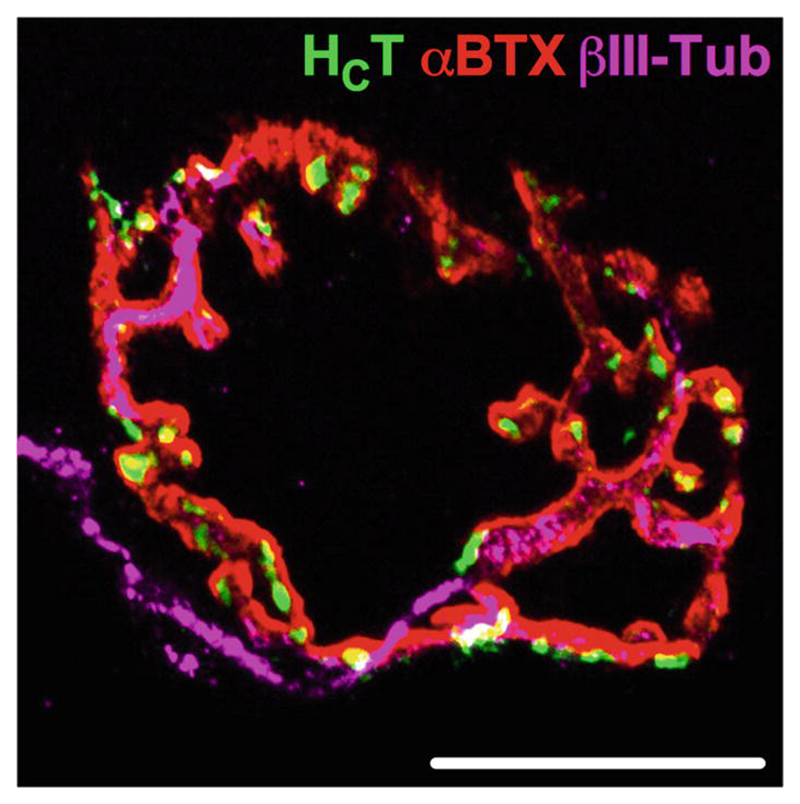
Tissues can be harvested and processed from imaged mice to provide a powerful, multipronged approach to understanding peripheral nerve diseases. After imaging, a variety of tissues, including sciatic nerves and muscles, can be collected and processed to provide extensive information on neuromuscular pathology in rodent disease models. Before being retrogradely transported, H_C_T-488 (green) is taken up into motor nerve terminals at the neuromuscular junction. This merged channel example comes from a 15 μm section of a fixed, freezing medium-embedded tibialis anterior muscle. Post-synaptic acetylcholine receptors are labeled with alpha-bungarotoxin (αBTX, red) and the motor neuron with anti-BIII-tubulin (BIII-Tub, magenta). Scale bar = 20 μm

Complementing an array of intravital techniques developed to assess in vivo axonal transport [15], we provide an in-depth, step-wise protocol to image bidirectional axonal transport of multiple cargo types within motor and sensory neurons of the sciatic nerve in live, anesthetized rodents. This technique has been used to confirm that several, but not all, mouse models of neurological disorders display early symptomatic deficits in axonal transport of signaling endosomes [17–20], indicating that disturbances in the process are not simply a biproduct of neurodegeneration and that impaired transport may play a causative role in only selected neu-ropathologies. Sciatic nerve exposure and imaging can also be coupled with a battery of transgenic fluorescent reporter strains [31, 32] in order to broaden the cargo types that can be imaged,as performed in ALS models [17, 33]. Furthermore, sciatic nerves can be dissected [34] post-imaging and processed for RNA and protein evaluation, which, when combined with neuromuscular (Fig. 8) [35] and sensory [36] phenotypic analyses in the same animal, can provide a powerful, multifaceted approach to understanding the pathological events underlying peripheral nervous system diseases. The method outlined in this chapter can be used to assess transport of multiple different cargoes in rodent disease models, as well as the impact of aging, potential therapeutics, and signaling molecules, which together will contribute to an improved general understanding of the mechanisms regulating axonal transport in healthy and diseased nervous systems.

## 4 Notes

1. Fluorescent probes are injected in excess to increase uptake by peripheral nerves, while BDNF is included to enhance this process [37]. However, we have confirmed that signaling endosomes can also be imaged using ten-fold less H_C_T (0.5 μg per muscle) and without BDNF coinjection.
2. Fluorescent H_C_T is taken up by both motor and sensory nerve terminals, whereas the anti-p75^NTR^ antibody preferentially locates to sensory neurons [6, 17]. p75^NTR^ is transported at a slower rate than H_C_T suggesting that motor neurons are capable of faster axonal transport of signaling endosomes [6, 17]. We recently used ChAT-eGFP mice to confirm that H_C_T-positive endosome transport is faster in motor neurons (ChAT^+^ axons) than sensory neurons (ChAT^–^ axons), and that the two peripheral nerve types can be differentiated in vivo by axon width [18]. We are thus able to assess and compare the axonal transport dynamics of signaling endosomes in motor and sensory neurons using only H_C_T.
3. The gastrocnemius and tibialis anterior muscles are coinjected to increase the number of peripheral nerve terminals exposed to fluorescent probe. The soleus muscle could also be coinjected. Alternatively, injections into single muscles may reveal distinct transport kinetics between axons innervating different muscles.
4. For all muscles, try to penetrate the fascia with a single injection as multiple punctures will lead to increased probe leakage. For this reason, it is also important to restrict damage to fasciae of adjacent muscles to avoid unwanted uptake of probes by other muscles; this is critical when comparing transport dynamics of axons innervating different muscles (*see* **Note 3**).
5. The tibialis anterior and soleus muscles are injected at a shallow angle to better accommodate the eye of the needle/micropipette due to the muscles being relatively thin. To maximize probe uptake, injections should be targeted to regions of muscle with the greatest density of nerve endings (e.g., motor end plates) [38, 39].
6. For soleus muscle and sciatic nerve injections, we recommend using graduated, glass micropipettes with microliter volume markers to aid accurate injection. As a guide, the microliter markings on the micropipettes we use are ≈5.4mmapart. Glass micropipettes can also be used to inject the probe into the gastrocnemius and tibialis anterior muscles, which we recom-mend for smaller mice. When carefully breaking the tip of the pulled micropipette, ensure that the solution can be pulled up and into the micropipette with ease. If the internal diameter of the opening of the micropipette tip is insufficient, the fluorescent probe can be forced up and out of the top of the pipette, limiting injection into the muscle.
7. Waiting 4–8 h from intramuscular injection before imaging is sufficient for a large number of fluorescent signaling endosomes to have reached the portion of exposed sciatic nerve. We have confirmed that H_C_T-positive signaling endosomes continue to be transported up to 96 h post-probe injection. From 4 to 96 h, the proportion of endosomes travelling in the anterograde direction increases, indicating that H_C_T can be used to analyze both retrograde and anterograde axonal transport.
8. Injection of additional fluorescent probes [40] (e.g., acidic organelle-targeting LysoTracker) is likely to permit in vivo imaging of additional cargo types. We have confirmed, using “MitoMice” [24], that TMRE is successfully loaded into mitochondria found within sciatic nerve axons.
9. Inject the sciatic nerve from the proximal side (i.e., closer to the hip than the knee) and try to eject the dye from above an axon bundle, rather than between the main sciatic branches. When the latter occurs, the dye can drain down to the distal end of the sciatic nerve and muscles limiting axonal dye penetration. Similar to muscle injections, a single piercing of the epineurium results in reduced dye leakage and better cargo labeling.
10. If injection of the sciatic nerve is proving difficult using **steps 5** and **6** of Subheading 3.3, it can help to insert one tip of fine curved forceps under the nerve (from the rostral end), adjacent and parallel with the left side of the magic tape strip, and use the other tip of the forceps not under the nerve, to support the micropipette. Doing this and tilting the forceps so that the free tip is slightly lower than the tip supporting the sciatic nerve allows for insertion of the micropipette tip beneath the epineurium on a similar plane to the nerve (approximating the 10° angle of the protocol described in **steps 5** and **6**). This reduces the chance of pushing the micropipette straight through the nerve and damaging axons. This procedure is best performed by rotating the mouse 90° counterclockwise so that the sciatic nerve extends away from you.
11. Advance the objective toward the stage until the lens has just contacted the glass coverslip. When you become accustomed to your microscope, you will know approximately how much further the objective needs advancing to focus on the sciatic nerve.
12. Sciatic nerve axons containing fluorescent signaling endosomes or mitochondria provide sufficient fluorescence signal and nonspecific labeling in surrounding tissues to focus on the nerve. First, advance the objective as outlined in **Note 11**—it does not matter if the focal plane is not yet perfect. Then move the stage in a systematic manner from left to right, down, right to left, down and continue until you find the region with the greatest amount of fluorescence. Once in this region, the focus can be readjusted until the nerve becomes visible. If cargoes prove difficult to find, then reposition the mouse on the stage, as this can uncover previously inaccessible, labeled axons (*see* **Note 14**).
13. When labeling of endosomes and mitochondria has worked well, fluorescence is visible with the naked eye using a 63× objective. However, this is not always the case, thus the computer software and confocal should be used to probe different axons, as modern confocal detectors are much more sensitive and able to detect low-level fluorescent signal. Opening up the pinhole and increasing the digital gain on the microscope software can also help with this. If cargoes still prove difficult to find, then reposition the mouse on the stage (*see* **Note 14**). If that fails, move the mouse back into the anesthetic chamber, reposition the anesthetic mask and start again. If the magic tape is cut too thinly, the surface area of the sciatic nerve that contacts the coverslip may also impair imaging. To restrict this, carefully drag the magic tape a small distance from its current position to increase the width of the magic tape underlying the nerve (i.e., away from the narrowest part); alternatively, cut a new, wider piece. We have successfully imaged transport in animals aged two weeks to 22 months. From our experience, fatter mice, which are often older animals, can sometimes be more difficult to image due to body fat restricting access to the sciatic nerve and due to fat within the sciatic nerve obscuring axons. It is recommended that the technique is thus first mastered on younger, leaner animals. Imaging transport in mice aged less than four weeks also becomes tricky due to their small size, and difficulties in maintaining stable anesthesia.
14. As the weight of the animal is the only thing holding the nerve in place, artifacts caused by breathing can impact the quality of images taken and thus the ability to analyze cargo transport kinetics; subtle breathing motions and sample drift have a large effect when imaging at high magnifications. The best way to counteract this is to observe the sample for 30–60 s to ascertain whether there are any obvious aberrations affecting the visibility of transport. If so, it is best to rearrange the mouse on the stage. Rotating slightly the animal onto its back often helps to reduce breathing movements as it puts more body weight on sciatic nerve to hold it in place. It is worth repositioning the mouse and persevering to get better quality images to simplify and improve postacquisition analysis. Additionally, anaesthesia depth can be adjusted by altering isoflurane concentration or oxygen flow rate to reduce larger breathing movements (e.g., gasping). As a last resort, tape can be used to attempt to restrict movement.
15. Multiple, distinct axons can be imaged simultaneously—we are frequently able to obtain data from three adjacent axons in a single video. When choosing an axon to image, an intermediate amount of cargoes is best; if the signal density is too high, it can be challenging to distinguish between neighboring cargoes across images, but when the axon is too sparsely populated, it can be difficult to generate sufficient data within the imaging time frame. This will partially depend on individual preference and how the data are to be analyzed (*see* **Note 17**), thus a variety of cargo densities should be imaged and tested in preliminary experiments. The situation is different for mitochondria because the percentage of mitochondria that is motile is much lower than that of signaling endosomes, making moving mitochondria easier to track when present in large numbers.
16. In our experience, TMRE, which is used to monitor active, polarized mitochondria, can bleach quickly and this makes finding axons with appropriate labeling difficult. As mentioned above, opening the pinhole and increasing the digital gain can help. In addition, first mastering the general technique of identifying individual axons in the exposed sciatic nerve using H_C_T, which does not bleach within the imaging time frame, will help to hone the skills required for rapid identification of axons containing TMRE-loaded mitochondria.
17. There is a trade-off between image quality and imaging time that requires balancing. For manual tracking of signaling endosomes, we image at a rate of every 2.5–3.5 s with line averaging 8, but when (semi)automated tracking is used, we image every 0.2–0.3 s with a single line average. As mitochondria move at a slower rate than endosomes, we tend to image mitochondria every 5–7 s and track them manually (Fig. 5, 6). A broad variety of cargo kinetic parameters can be analyzed [15].
